# Application of Marine Natural Products against Alzheimer’s Disease: Past, Present and Future

**DOI:** 10.3390/md21010043

**Published:** 2023-01-05

**Authors:** Di Hu, Yating Jin, Xiangqi Hou, Yinlong Zhu, Danting Chen, Jingjing Tai, Qianqian Chen, Cui Shi, Jing Ye, Mengxu Wu, Hong Zhang, Yanbin Lu

**Affiliations:** 1 Collaborative Innovation Center of Seafood Deep Processing, Key Laboratory of Aquatic Products Processing of Zhejiang Province, Institute of Seafood, Zhejiang Gongshang University, Hangzhou 310012, China; 2 Hangzhou WeChampion Biotech. Inc., Hangzhou 310030, China; 3 Zhejiang Chiral Medicine Chemicals Co., Ltd., Hangzhou 311227, China

**Keywords:** Alzheimer’s disease, neurodegenerative disease, therapeutic, pathogenesis, marine natural products

## Abstract

Alzheimer’s disease (AD), a neurodegenerative disease, is one of the most intractable illnesses which affects the elderly. Clinically manifested as various impairments in memory, language, cognition, visuospatial skills, executive function, etc., the symptoms gradually aggravated over time. The drugs currently used clinically can slow down the deterioration of AD and relieve symptoms but cannot completely cure them. The drugs are mainly acetylcholinesterase inhibitors (AChEI) and non-competitive N-methyl-D-aspartate receptor (NDMAR) antagonists. The pathogenesis of AD is inconclusive, but it is often associated with the expression of beta-amyloid. Abnormal deposition of amyloid and hyperphosphorylation of tau protein in the brain have been key targets for past, current, and future drug development for the disease. At present, researchers are paying more and more attention to excavate natural compounds which can be effective against Alzheimer’s disease and other neurodegenerative pathologies. Marine natural products have been demonstrated to be the most prospective candidates of these compounds, and some have presented significant neuroprotection functions. Consequently, we intend to describe the potential effect of bioactive compounds derived from marine organisms, including polysaccharides, carotenoids, polyphenols, sterols and alkaloids as drug candidates, to further discover novel and efficacious drug compounds which are effective against AD.

## 1. Introduction

Dementia is an umbrella term for a particular group of symptoms. The characteristic symptoms of dementia are difficulties with memory, language, problem-solving, and other thinking skills that affect a person’s ability to perform everyday activities. Alzheimer’s disease (AD) is the most common cause of dementia, accounting for an estimated 60% to 80% of cases [1]. AD is associated with age and characterized by losing neuronal structure and function gradually [2]. Also, with the population growing and life expectancy increasing, the prevalence of AD continues to rise [3]. The symptoms of AD are mainly associated with progressive memory impairment, aphasia, purposeful complex activity ability. At the same time, Alzheimer’s sufferers have an inability to distinguish previously familiar objects through specific senses, impaired visuospatial abilities, impaired abstract thinking and numeracy, and changes in personality and behavior.

Over time, symptoms will worsen and can even be life-threatening. The pathological sequence of AD starts in the center temporal lobe firstly, which is responsible for memory, and then progresses to the regions of the frontal, temporal, parietal, motor, sensory, and subcortical areas. The neuropathology of AD is complex and not yet fully understood [4]. The 2011 National Institute on Aging and Alzheimer^’^s Association (NIA-AA) guidelines defined three phases of AD: preclinical AD, MCI (symptomatic predementia) and dementia [5]. Preclinical AD, the earliest phase from normal cognition to AD dementia is characterized by the fact that daily life and work are basically not affected and one’s ability to live independently is relatively complete (lasting about three years, accompanied by mild cognitive difficulties). The most notable deficits are memory loss (e.g., it is hard to recall newly learned knowledge, unable to communicate new messages or there are difficulties in semantic memory). Certain difficulties with coordination and planning may arise when doing some delicate motor tasks (for instance, difficulties in writing, painting or dressing also occur at this stage) [6]. Even when people walk on familiar roads, they can also lose their way [7]. In the middle stage of the disease (the MCI stage), patients have the inability to live independently and will almost die from various accidents and complications; this phase lasts about two years. Changes in behavior are more pronounced at this stage, and the patient begins to no longer recognize their family and other close people [8]. Approximately 30% of patients are at risk of urinary incontinence [9], along with muscle mass decreases, inactivity, and becoming bedridden. However, the ability to receive and send emotional signals is still there [10]. In the advanced stage of the disease, patients totally lose the ability to take care of themselves and their behaviors deteriorate; most of them pass away due to various complications within one to two years.

AD has become a serious public health issue with high medical costs and no cure. Most cases appear in people over 65 years of age, and the global morbidity is about 6%, with women having a higher morbidity rate than men. The Alzheimer’s Disease Society International’s Association published a report entitled “The Global Impact of Dementia 2013−2050”, which showed that 44 million people are ill with dementia, a 17% raise from 2009, and that the number of people worldwide with dementia is almost doubling every 20 years. By 2030 and 2050, 76 million and 135 million cases of dementia will be found around the world. China has the highest prevalence rate with about 10 million people of dementia in the world and a yearly increase of 30 percent. In China, the prevalence rate is 5% for those over 65 years old and 30% for those over 85 years old. With the fast aging of China’s population, the number of people with dementia in China will exceed 20 million by 2050, 10% of the elderly over 75 years old will have cognitive impairment, and one third of the elderly over 85 years old will have cognitive impairment [11].

In view of the increasing number of patients with AD, researchers have never stopped exploring therapeutic drug development. In recent years, many researchers have found that in the marine environment there are a large number of unique and different structures, including polysaccharides, carotenoids, polyphenols, sterol and alkaloids, which have biological and pharmacological activities [12]. Marine ecosystems cover more than 70% of the earth’s surface, accounting for about half of the global biodiversity [13]. Marine bioactive compounds have unique biological activities due to their chemical properties that are not found in terrestrial products [14]. Therefore, people are increasingly studying these resources to explore drugs that can treat human diseases [15,16]. This review briefly introduces the related pathology of AD and comprehensively expounds the drug potential of marine compounds which is found to have the potential to treat AD based on the therapeutic targets of AD. The purpose of this review is exhibiting the great potential of marine natural products, and also providing the direction for the development of new therapeutic drugs for AD.

## 2. Pathogenesis of Alzheimer’s Disease

**Figure 1:**
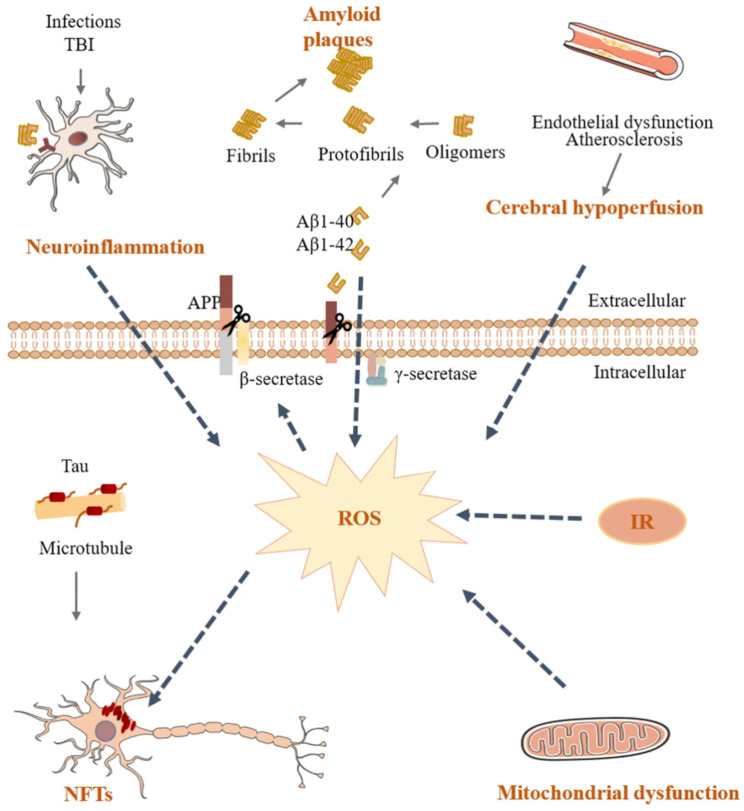
Key factors in the pathogenesis of AD. Aβ, amyloid-beta; AD, Alzheimer’s disease; APP, amyloid precursor protein; IR, insulin resistance; NFT, neurofibrillary tangle; OS, oxidative stress; ROS, reactive oxygen species; TBI, traumatic brain injury. (Re-produced with permission from [20], Springer Nature, 2020.)

According to the age of onset, Alzheimer’s Disease (AD) can be divided into two types: early onset (EOAD) and late onset (LOAD). EOAD has certain heritability, accounting for a small number of all Alzheimer’s disease. The majority of patients have LOAD. Age is the greatest risk factor for acquiring AD. In adults older than 85, the prevalence of AD is more than one in three [17,18,19]. At present, there is no final conclusion on the pathogenesis of Alzheimer’s disease. However, there is no doubt that neuroinflammation, extracellular plaques, and intracellular neurofibrillary tangles (NFTs) are key pathological trademarks of this illness. Key factors in the pathogenesis of AD are shown in Figure 1 [15]. There are several pathogenesis concerning AD as described below.

### 2.1. Amyloid Cascade Hypothesis

The first hypothesis is the amyloid cascade. This hypothesis concludes that Aβ aggregation as an early event in neurodegeneration occurs independently prior to tangles formation, followed by microglia and astrocyte activation, neuroinflammatory responses, oxidative stress, and other cellular molecular events [21]. The pathogenesis of amyloid protein starts from the change and cleavage of amyloid precursor protein (APP). β-secretase (BACE1) and γ-secretase change and split APP to produce insoluble Aβ raw fiber. Then, Aβ aggregation form the plaques. On the one hand, Aβ oligomerization spreads to the synaptic gap that interferes with synaptic signal transmission. On the other hand, this polymerization leads to the activation of kinases, which promotes hyperphosphorylation of microtubule correlation tau protein. Then, hyperphosphorylated protein is polymerized into insoluble NFT. After aggregation of plaque and NFT, microglia around the plaque gathered. This promotes microglial activation and local inflammatory response and aggravates neurotoxicity [22]. From the above process, it can be seen that Aβ is like a trigger button in the process of the disease, and it has been proposed that Aβ is a trigger target for pathological processes. Therefore, Aβ is a major therapeutic target [23]. Perhaps this is why many people regard it as the primary drug target.

### 2.2. Neuroinflammation Hypothesis

Another hypothesis is the neuroinflammation hypothesis, which is modified from the above-mentioned amyloid hypothesis. Based on this theory, the pathogenesis of AD is mainly related to a series of activities of microglia in the immune system of the central nervous system (CNS) in which that microglia continuously activate pro-inflammatory cells through the transduction of pro-inflammatory signals [24]. At this point, the microglia can’t adjust the impairment of anti-inflammatory cytokine and lipid dielectric, causing damage to the nerve degeneration and neuronal metabolites, which brings more inflammation and excessive phosphorylated tau protein increased. Long term activation of the immune response has been shown to worsen AD pathology, possibly as a result of persistent activation of microglia in a feedforward loop (termed reactive microgliosis). This leads to the accumulation of Aβ and the persistent single-shot of pro-inflammatory cytokines that start to damage neurons [25]. Many elderly people have amyloid plaques in their brains that never develop into Alzheimer’s disease. Amyloid protein accumulation itself is not enough to cause dementia. The research results of Pascoal et al. show that it is the interaction between neuroinflammation and amyloid pathology that releases the spread of tau protein, which ultimately leads to extensive brain damage and cognitive impairment [26]. Now, neuroinflammation is established as a feature of Alzheimer’s.

### 2.3. Ca^2+^ Hypothesis

The next hypothesis is the Ca^2+^ hypothesis. Ca^2+^ dysregulation is a common and pervasive pathophysiological phenomenon in AD. Zhong et al. [27] hypothesized that N-methyl-D-aspartate receptor subunit (GluN3A) is essential for sustained Ca^2+^ homeostasis and its deficiency is a causative factor in AD. By examining cellular, molecular and functional changes in adult/senescent GluN3A knockout (KO) mice, they concluded that chronic ‘degenerative excitotoxicity’ can lead to sporadic AD, and GluN3A is the main pathogenic factor, a lifelong moderate but sustained Ca^2+^ overload is a causal pathogenic mechanism of sporadic AD. Therefore, GluN3A may be an amyloid-independent therapeutic target [27].

### 2.4. Tau Hypothesis

The tau hypothesis is based on the notion that tau protein’s hyperphosphorylation leads to NFTs, which is one of the chief pathological conditions of AD [28]. Clinical studies have found that the quantity of hyperphosphorylated tau protein and NFTs in the cerebrum of AD patients is positively associated with the degree of clinical dementia. That is to say the higher number of hyperphosphorylated tau proteins and NFTs, the more severe clinical and stupid condition. The tau protein is encoded by the microtubule-associated protein tau (MAPT) gene on chromosome 17. MAPT produces a monolithic hydrophilic protein, which is enriched in large, naturally unexpanded regions of developing and mature neuronal axons [28]. The tau protein exists in two isomers, namely the 3-repeat sequence (3R) and 4-repeat sequence (4R), in which the 3R tau protein mainly occurs during development and 4R tau protein is mainly produced in adulthood. These two isomers maintain a balanced proportion (1:1) in the grow-up of the cerebrum. Disturbance of the proportion between 3R and 4R may lead to AD and other diseases. It is also mentioned in the amyloid hypothesis that Aβ polymerization activates kinases, which promotes hyperphosphorylation of microtubule correlation tau protein [22]. So, inhibition of kinase activity may an effective way to control tau hyperphosphorylation. Some related studies have demonstrated that reducing tau phosphorylation by inhibiting tau kinases can restore tau-dependent long-term potentiation (LTP) deficits and attenuates synaptic loss in tau transgenic mice [29]. The novel role of pathological tau protein in disease progression will provide more directions for the search of alternative disease mechanisms and related treatment strategies in the field of Alzheimer’s disease.

### 2.5. Cholinergic Hypothesis

The pathogenesis of AD is usually related to the decrease of neurotransmitter levels, such as serotonin, norepinephrine, dopamine, acetylcholine, etc. Acetylcholine (ACh), which is closely associated with the formation and storage of human’s memory ability, is an important neurotransmitter in the human cerebrum. Decreasing the level of Ah can directly lead to cell damage in the basal nucleus, temporal lobe, and parietal lobe, thereby reducing the level of serotonin and intensifying the development of NFT [30]. The theory states that AD-related psychiatric symptoms arise are related to the impairment of cholinergic neurons and dopaminergic transmission [31,32]. ACh regulates many key functions of the CNS by activating cell-surface receptors known as muscarinic acetylcholine receptors (M1-M5 mAChRs), which are co-expressed with D1 dopamine receptors in a specific subset of striatal projection neurons. It has been proved clinically that selective M1 agonists can improve the cognition of AD patients and reduce Aβ in cerebrospinal fluid level [33]. Besides, studies on M4 gene knockout mice showed that M4 gene deletion increased dopamine efflux in the nucleus accumbens of mice, which confirmed the physiological relevance of M4 mAChR subsets in regulating dopamine-dependent behavior and indirectly verified this hypothesis [34]. Currently, some of the drugs are used to treat Alzheimer’s disease and target acetylcholinesterase.

### 2.6. Glutamate Hypothesis

Finally, the glutamate hypothesis is based on the truth that cognitive disability in AD is closely related to synaptic plasticity, and that N-methyl-D-aspartate receptor (NDMAR) plays a key role [35]. Excitatory glutamatergic neurotransmission through the NMDAR is crucial for neuronal synaptic plasticity and staying alive. However, it is a potential mechanism of AD neurodegeneration that NMDAR overacts excitotoxicity and promotes cell death [36]. As a kind of AD drug, it works by inhibiting NMDAR activation based on this hypothesis exactly.

## 3. Currently Approved Drugs for Alzheimer’s Disease

Currently, it is reported that about 24 million people worldwide are living with AD. Alzheimer’s disease has become a public health problem, but before June 2021, there were only two types of medicines have been permitted to treat AD patients, including cholinesterase enzyme inhibitors (naturally derived, synthetic and mixed analogs) and NMDAR antagonists [37]. The pathway of acetylcholine-producing cells in AD patients is disrupted, and the role of acetylcholine inhibitory enzymes is to prevent choline enzyme from breaking down acetylcholine, thereby increasing the level of acetylcholine in the synaptic [38]. NMDAR antagonists prevent excessive activation of NMDAR, thereby preventing calcium influx and avoiding cell death and synaptic dysfunction cause by increased calcium ion concentration [36]. Although these two types of drugs have therapeutic effects on Alzheimer’s disease, they can only relieve symptoms and cannot achieve the effect of cure and prevention. In June 2021, the US Food and Drug Administration officially approved aducanumab for the treatment of Alzheimer’s disease. This is the first therapeutic drug targeting the potential pathophysiology of disease since 2003. Besides, on November 2, 2019, Sodium Oligomanne Capsules (GV-971) was approved by the State Drug Administration of China for marketing, which is the first new drug for Alzheimer’s disease that targets brain gut axis in China and the world. On April 8, 2020, Shanghai Green Valley Pharmaceuticals, China received the formal decision letter from the US Food and Drug Administration (FDA) on the Investigational New Drug (IND) application for the GV-971 international multi-center Phase III clinical study [39]. However, the manufacturer, Green Valley Pharmaceutical, announced the early termination of the international multicenter phase III clinical study of this drug on May 13, 2022, due to insufficient research funds. Table 1 presents several related information about drugs currently used for AD treatment.

### 3.1. Acetylcholinesterase in-jibtor (AChEI)

#### 3.1.1. Tacrine

A common kind of drugs for curing of mild to moderate AD and related dementias is cholinesterase inhibitors (ChEIs) [40]. Tacrine was the first cholinesterase inhibitor medicine to be used, which was first synthesized in the 1930s. Tacrine has been used in patients with AD since the 1980s, having been approved by the FDA in 1993 and discontinued in 2013 [38]. Tacrine has inhibitory effects on both acetylcholinesterase (AChE) and butyrylcholinesterase (BChE), but was stopped due to its side effects such as gastrointestinal side effects and hepatotoxicity in patients.

#### 3.1.2. Donepezil

Donepezil is a second-generation reversible AChEI drug that is also used to treat patients with mild to moderate AD [41]. Its mechanism of action is that donepezil is reversibly combined with acetylcholinesterase so that it has inhibitory effect to hydrolysis of acetylcholine. To do that, it achieved the effect of increasing the concentration of acetylcholine at the synapse in the end. However, it should be noted that donepezil is only used to treat AD symptoms, such as improving cognition and behavior, but does not change AD progression [44].

#### 3.1.3. Rivastigmine

Rivastigmine is a reversible dual inhibitor of AChE and BChE in a slow treatment speed. There are two ways of administration: oral and transdermal. It is approved by FDA that oral dosage form of rivastigmine is used for treating mild to moderate AD. The optimum therapeutic dose is 6–12 mg/day [42]. Gastrointestinal side effects can also occur with this drug (for better tolerance it is recommended to take it with food, twice a day) [45]. Transdermal patch, another mode of administration, is a paper-thin, waterproof matrix patch, which has the advantages of easy administration and reduced tablet burden for patients taking combination drugs and patients with dysphagia. Appears to have adverse skin reactions, most commonly irritant contact dermatitis, which is usually mild, transient, and can be controlled with topical treatments (such as topical corticosteroids) [46].

#### 3.1.4. Galantamine (GAL)

Galantamine, as a standard first-line drug, is used for treating mild to moderate AD. GAL is a selective tertiary isoquinoline alkaloid with a dual mechanism of competing with acetylcholine receptor α-subunit to prevent it combine with acetylcholine inhibitors and to activate acetylcholine [38]. Adverse reactions that may occur with this drug include weight loss, diarrhea, loss of appetite, nausea, vomiting, dizziness, headache, gastrointestinal bleeding, etc. [46].

### 3.2. NMDAR Antagonists

Memantine, which is an antagonist of the NMDAR, is an FDA-approved prescription drug for the treatment of moderate-to-severe Alzheimer’s disease [37]. The drug reportedly targets extrasynaptic NMDARs preferentially [47]. Adverse reactions that may occur while taking this drug include constipation, diarrhea, confusion, dizziness, and headache [46].

### 3.3. Aducanumab

**Figure 2:**
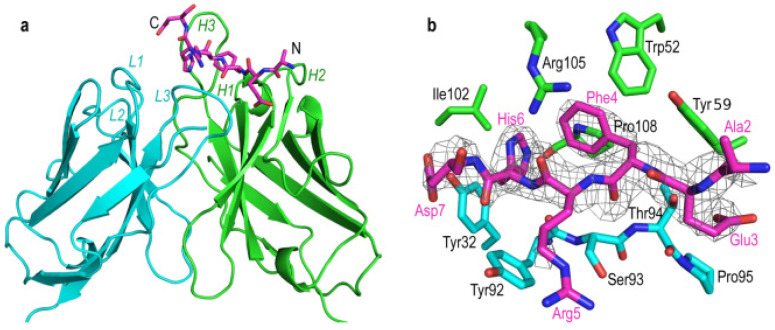
The structure of AduFab with bound Aβ1-11 peptide. (**a**) Cartoon representation of AduFab showing heavy chain in green, light chain in cyan, and Aβ1-11 peptide in magenta, with nitrogen and oxygen atoms displayed in blue and red, respectively. L1 to L3 and H1 to H3 indicate the CDRs in the light and heavy chain, respectively. (**b**) Detailed view of the binding interface between AduFab and the Aβ1-11 peptide, with key interface residues of AduFab within 4 Å of the Aβ peptide shown and labeled. An omit electron density map contoured at 3.0 σ is shown as mesh and superposed on the Aβ peptide. (Re-produced with permission from [48], Springer Nature, 2018.)

Aducanumab, with the structural formula shown in Figure 2 [48], is one kind of monoclonal antibody of human immunoglobulin γ (Ig G)1. By recognizing Aβ epitopes, Aducanumab acts on soluble and insoluble Aβ, reduces its content, and has low affinity for Aβ monomers [48]. The most common adverse reaction of the drug in clinical trials is brain edema or hydrocephalus. The launch of aducanumab was also controversial, with only the EMERGE (NCT02484547) [49] Phase III global trial showing a significant reduction in Aβ in AD patients and a significant improvement in the CDR-SB score, a measure of cognitive function. Therefore, the FDA accelerated the approval of aducanumab but also required phase IV trials to further verify the efficacy and safety of aducanumab.

### 3.4. Sodium Oligomanne Capsules (GV-971)

Sodium Oligomanne Capsules (GV-971) is a low molecular acid oligosaccharide compound prepared from the extract of marine brown algae [50]. In non-clinical studies, the mechanism of action of GV-971 is to decrease peripheral inflammation that may aggravate neuroinflammation by regulating the gut microbiota, thereby reducing the neuroinflammation in the brain. GV-971 combines with Aβ in direct to decreases Aβ deposition in the brain [51,52,53]. This drug is mainly used to treat patients with mild to moderate AD and may cause skin rash, abdominal pain, and other adverse reactions in patients.

### 3.5. Combination Drug Therapy

Alzheimer’s disease is a chronic disease, and patients usually require long-term medication. For patients on long-term medication, therapeutic effects may be limited (in a fixed dose regimen) due to neurobiological adaptation and drug tolerance, promoting the need increase the dose further. Therefore, lowering the drug dose as much as possible while still achieving the therapeutic effect can reduce the possibility of induced toxicity. Combination drug therapy may be a good method [54]. Hafsa Amat-ur-Rasool et al. performed in vitro experiments on the inhibitory effect of anticholinesterase combined drugs on AChE protein, the inhibitory effect of AChE in differentiated neurons, and the cytotoxicity of neuronal cells. Two-drug combinations of berberine and tacrine (BerTac), berberine and galantamine (BerGal), and tacrine and donepezil (TacDon) all produce synergistic AChE inhibition results. Donepezil and galantamine (DonGal) have a synergistic effect on human AChE, but have an antagonistic effect on tcAChE. After the combination of the two drugs was applied to neuronal cells, BerTac, BerGal, DonGal and donepezil and berberine (DonBer) all showed synergistic inhibition of AChE. BerGal produced the most potent synergy, reducing the total drug dose by 72% [55]. In China, there are also a few reports on the use of sodium oligomannate or memantine combined with donepezil in the treatment of mild Alzheimer’s disease cases.

Currently, there is no specific drug that can treat AD. Therefore, the development of new specific drugs is an urgent problem to be solved. The huge number of marine organisms can produce a large number of natural products with unique structures, some of which have neuroprotective effects and may be used for AD treatment. At the same time, the successful marketing of GV-197, which was made from marine brown algae extracts in China, shows us the bright prospect of marine biological ingredients as the treating drugs of AD.

## 4. Anti-AD Marine Natural Products

Marine natural products have increasingly allured great scientific interest since they can show significant biological activity 10 times higher than terrestrial organisms [14]. Through the study of the biological functions of marine natural products, it has continuously proved its potential in the biomedical neighborhood, including anti-thrombotic, anti-coagulant, anti-inflammatory, anti-hypertension, anti- diabetes, heart protection, and neuroprotection effects [56]. A large and growing literature has investigated that marine natural products have great potential against AD due to their chemical compounds, including polysaccharide, polyphenols, sterol, carotenoids, diterpenoids, alkaloids, amino sulfonate, marine toxins and macrolide-type compounds [14], as summarized in Table 2. In the process of extracting marine compounds and developing new drug molecules, several key parameters help to determine the overall safety of precursor drugs, including absorption, distribution, metabolism, excretion, and toxicology (ADMET). We have listed the information related to trans-blood brain screen, skin permeability, and drug toxicity of some marine compounds in Table 3.

### 4.1. Polysaccharides

Polysaccharides are polymeric carbohydrates formed by the combination of sugar chains and glycosidic bonds with at least ten or more monosaccharides with high molecular weight [73]. So far, more than 300 natural polysaccharide compounds have been founded in the literature, such as fucoidan, chitosan, mannan, and seaweed polysaccharide, among which the fucoidan and chitosan have the potential to treat AD.

**Figure 3:**
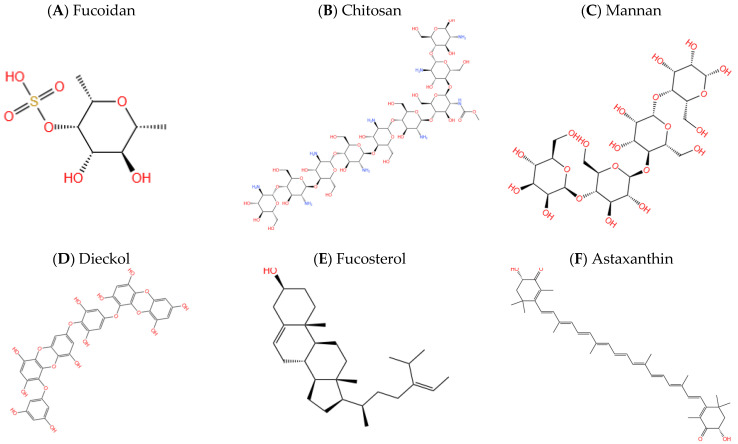

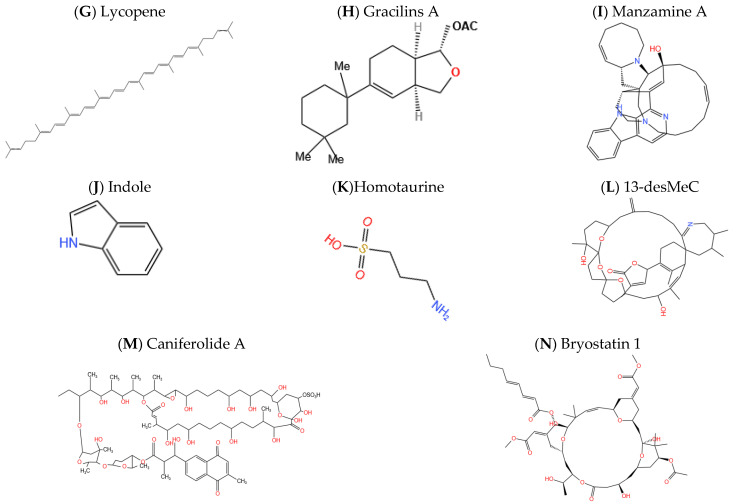
Chemical structures of marine molecules with neuroprotective effects against Alzheimer’s Disease: fucoidan (**A**), chitosan (**B**), mannan (**C**), dieckol (**D**), fucosterol (**E**), astaxanthin (**F**), lycopene (**G**), gracilins A (**H**), manzamine A (**I**), indole (**J**), homotaurine (**K**), 13-desMeC (**L**), caniferolide A (**M**) and bryostatin 1 (**N**).

Fucoidan (Figure 3A) is a sort of complicated polysaccharide compound, which separated from brown seaweed and some marine invertebrates [74]. It mainly contains L-fucoses and sulfate groups [75], which has extensive pharmacological actions, such as anti-virus, oxidation resistance, anti-inflammatory, and anticoagulant, improving Aβ1-40 induced memory impairment, etc. [75,76]. Fucoidan consists of two chain structures, one with α- (1→3) connected L-fucose residues as the chain and the other with alternating α- (1→3) and α- (1→4) connected L-fucoses that may be sulfate substituted on C-4 [3]. It can block the activation of the enzymes caspase-9 and caspase-3 by β-amyloid protein (Aβ), which plays an important role in apoptosis processes. It is precisely due to the fact that it can inhibit apoptosis that the compound also has a significant effect in terms of neuronal death suppression. A fucoidan treatment has been found capable of suppressing the intracellular Ca^2+^ responses of neurons [77]. The superfluous accumulation of intracellular Ca^2+^ may go in front of the wound and put the neurons to death. Studies have indicated that high attention of fucoidan can cause and prevent the dying of dopaminergic neurons in vivo and in vitro models [57,78]. Fucoidan can reduce the neurotoxicity of β-amyloid protein, since it can reduce toxicity by reducing the inhibition of β-amyloid protein on protein kinase C (PKC) phosphorylation [57,79]. So, it can produce neuroprotective effect. In addition, it was found that fucoidan could reduce neuronal damage in AD mouse model [80]. The compound can raise mitochondrial activity and reduce the release of lactate dehydrogenase (LDH) and reactive oxygen species (ROS) for enhancing the neuroprotection. It can enhance the expression of anti-apoptotic-protein Bcl-2 and lessen the expression of Pro apoptotic protein Bax for dramatically inhibiting neuronal apoptosis [81].

Chitosan (Figure 3B), called deacylated chitin, is a natural polysaccharide that can be obtained from marine crustaceans or in the cell walls of some fungi. Chitosan is a polymer of β- (1→4)-D-glucosamine or a copolymer with N-acetyl-D-glucosamine, which is formed by enzymatic N-deacetylation of chitin on fungal cell wall by chitin deacetylase [82]. Chitosan is difficult to be used in food and biomedical fields due to its poor solubility [83]. Therefore, researchers mostly convert chitosan to chitosan oligosaccharides (COS) that are easily soluble in water [84]. Chitosan and COS have been proven to plays a significant part in oxidation resistance and antibacterial, anti-apoptosis, and immune regulation [58,85]. Khodagholi et al. [86] studied the effect of chitosan on NT2 neuron cells induced by H_2_O_2_ and FeSO_4_, and found that chitosan treatment could inhibit the death of NT2 neuron cells induced by oxidative stress. Moreover, the formation of Aβ in NT2 neurons pretreated with chitosan was significantly lower than that in control cells. The results showed that the Aβ level could controlled by treatment with this chitosan, suggesting that chitosan had a certain protective effect on AD. Yoon et al. synthesized COS derivatives with different substituents. Among the three COS derivatives, Diethylamino-ethyl COS (DEAE-COS) has the strongest AChEIs activity [87]. The results showed that DEAE-COS was identified as a competitive AChEIs and that chitosan and its derivatives could inhibit the activity of acetylcholinesterase and had a potential neuroprotective effect on neuronal disorders [58]. Numerous studies have shown that it can restrain the formation of amyloid-β and the activities of acetylcholinesterase. Also, it has the peculiarity of anti-neuroinflammation and anti-apoptosis properties [58,88]. So far, the neuroprotective effects of chitosan and its derivatives have mostly been observed in vitro [89]. Therefore, further studies are needed to investigate their activity in mouse model systems and/or human subjects. Taken together, these results revealed the potential of chitosan and its derivatives as potential therapeutic candidates for neurodegenerative diseases, and they hold great promise in future drug development.

Mannan (Figure 3C) is a polysaccharide widely existing in nature, which exists in plants, marine bacteria, yeast, etc. It has the characteristics of high water solubility, stability, and viscosity [90]. Mannan is composed of monosaccharide D-mannose linked by β-1,4-mannoside linkage [91]. Liu et al. [59] investigated the effect of the hydrolytic product of mannan, mannan oligosaccharides (MOS) on 5xFAD-Tg mice, a classic transgenic AD mouse model. It was found that after eight weeks of MOS (0.12%, w/v in the drinking water) oral administration, cognitive function and spatial memory were significantly improved and behavioral disorders were reduced in mice. In addition, immunofluorescence staining showed that MOS treatment improved the neuronal morphology of AD-affected mice brains, and greatly reduced the accumulation of Aβ in the cerebral cortex and hippocampus. Importantly, MOS treatment reduced the overexpression of tumor necrosis factor-α (TNF-α) and interleukin 6 (IL-6) in the brains of AD mice, effectively alleviated neuroinflammation and oxidative damage in the brains of AD mice [59]. Therefore, further clinical trials are needed for evaluation to explore the therapeutic effect of chitosan on AD patients.

### 4.2. Polyphenols

Polyphenols are a sort of neuroprotective compound that consist of aromatic ring and one or more phenolic rings in natural plants [92,93]. Algae are rich in polyphenols (especially phloroglucinol, a kind of phlorotannins which has been proved to have many biological activities) [94]. An experiment was conducted to investigate the effect of brown algae polyphenols on scopolamine-induced memory impairment in mice. The results showed that phlorotannins (50 or 100 mg/kg) orally supplemented for six weeks improved scopolamine-induced memory impairment in behavioral tests [93]. Phlorotannins decreased acetylcholinesterase activity in the brain, and significantly reduce lipid peroxidation levels but increased glutathione levels and superoxide dismutase activity [93]. In addition, phlorotannins upregulate the expression levels of brain-derived neurotrophic factor (BDNF), phosphorylated extracellular signal-regulated kinase (ERK), and cyclic AMP-response element-binding protein (CREB), which influence cholinergic dysfunction and memory deficit [95,96]. It has been found that phlorotannins including dieckol, eckol, and 8,8′-bieckol can inhibit the BACE-1 enzyme.

Currently, drugs used to treat AD work mainly by inhibiting acetylcholinesterase, so the discovery that phlorotannins including dieckol, eckol, and 8,8′-bieckol have an inhibitory effect on BACE-1 enzyme may improve medication and treatment for AD patients [60,97]. Liu et al. evaluated the neuroprotective effects of three polyphenols, including 8,8’-bieckol, dieckol, and eckol, on Aβ25-35-mediated cytotoxicity in PC12 cells. Of these, dieckol (Figure 3D) showed the greatest protective effect, although both had been shown to inhibit inflammatory responses by inactivating the NF-κB pathway [98]. Dieckol has been found to have a powerful inhibitory effect on amyloid-β peptide (Aβ) accumulation in and out of the cell. Yoon et al. [60] demonstrated for the first time that dieckol regulates APP proteolysis and Aβ production by regulating the phosphoinositide 3-kinase (PI3K)/protein kinase B (Akt)/Glycogen synthase kinase 3β (GSK-3β) signaling pathway. In addition, the addition of PI3K inhibitor LY294002 counteracted all of the effects of dieckol, suggesting that Akt/GSK-3β is the main pathway mediating Aβ production by Swedish mutant APP over-expressed Neuro-2a (SweAPP N2a) cells. The current findings support a better understanding of the important role of dieckol in the prevention of AD and its potential as a promising source of anti-AD drugs [60,99].

### 4.3. Sterols

Marine sterols are sterol compounds produced by organisms in ocean. They are the commonest natural organic compounds in the ocean (and are mostly from marine algae). Marine sterols such as fucosterol (Figure 3E) have the capability to lessen the content of cholesterol in the blood, leading to the tubular formation of cholesterol in the body [100]. However, the out of balance of cholesterol homeostasis will lead to inflammation, which is related to the pathobiology of neurological diseases [61]. Furthermore, fucosterol exhibited moderate anti-AChE activity by Ellman’s method [101]. So, it may have an improvement effect on the symptoms of AD. In addition, sterols have anti-inflammatory effects in macrophages and can prevent LPS- or Aβ-mediated neuroinflammation [102]. It is found that sterols have anti-BACE1, and Aβ aggregation inhibitory activities [62]. Castro-Silva et al. evaluated the AchE inhibitory activity of sterols isolated from Sargassum seagrass extracts in vitro and in silicon. The sterol not only has a higher affinity for AchE compared to the positive control, but is also a non-competitive human acetylcholinesterase (hAChE) inhibitor, which differs from the stabilizing effect of galantamine (competitive hAChE inhibitor) [103]. Marine sterols can reduce cell apoptosis and inhibit neuroinflammation by regulating brain-derived neurotrophic factor (BDNF, synaptic growth factors associated with memory and learning), nuclear factor erythroid 2-2-related factor 2 (Nrf2), and nuclear factor kappa-light-chain-enhancer of activated B cells (NF-κB) signaling systems [98,104].

### 4.4. Carotenoids

Carotenoids are a kind of significant pigments in natural world, which extensively exist in animals, plants, fungus, and algae (the vast majority of which come from algae) [64,105]. They are formed by linking isoprenoids units and exist in various colors, including fucoxanthin, lycopene, lutein, astaxanthin (Figure 3G), etc. In fact, fucoxanthin (Figure 3F), which was abundant in brown seaweed, was isolated from *S. horneri* and evaluated for its effect on cognitive impairment in vivo and its ability to inhibit some key enzymes in vitro by Lin et al. [106]. It was concluded that fucoxanthin could effectively reverse scopolamine-induced cognitive impairment in mice, significantly increased choline acetyltransferase (ChAT) activity and BDNF expression, and decreased AChE activity in scopolamine treated mice, suggesting that fucoxanthine has the potential to enhance cognition [12,106]. Another study explored the direct effect of fucoxanthine on Aβ assembly and evaluated the effect of fucoxanthine on the cognitive performance of Aβ oligomer-treated mice [107,108]. The results demonstrated that fucosanthin may effectively reverse cognitive impairment by inhibiting oxidative stress and upregulating the expression of BDNF and ChAT in Aβ1-42 oligomeric-treated mice, suggesting that fucosanthin reduces Aβ neurotoxicity in vivo, which may be useful for prevention of AD [107]. In addition, fucoxanthin also showed anti-inflammatory and antioxidant effects [63]. Lycopene (LYC,Figure 3G), a fat-soluble carotenoid, was found to have inhibitory effects on LPS-induced memory loss, to inhibit the phosphorylation of lipopolysaccharide (LPS)-treated BV2 microglia MAPKs and NF-κB, and activate the Nrf2 signaling pathway in the Morris water maze test of mice, suggesting that LYC may be a preventive strategy for neuroinflammatory related diseases such as AD [65]. In vitro and in vivo studies were conducted in rat models to investigate the effects of astaxanthin on lipopolysaccharide LPS-induced inflammatory responses [109]. The results showed that the anti-inflammatory effect of astaxanthin (100 mg/kg) was higher than that of commonly used anti-inflammatory drugs (10 mg/kg), and that LPS-fed mice treated with astaxanthin showed a dose-dependent anti-inflammatory effect. Astaxanthin plays an anti-inflammatory role by inhibiting the production of (nitric oxide) NO, prostaglandin E2 (PGE2), TNF-α and interleukin-1β (IL-1β) [64]. There are currently clinical trials (NCT05015374) of astaxanthin in patients with AD taking astaxanthin or a placebo. The trials are expected to end in 2024, after which the possible benefits of astaxanthin on Alzheimer’s disease will be examined by studying patients’ mental status, cognitive ability, and clinical dementia scores. Overall, the results of the neuroprotective effects of carotenoids in vitro and in vivo are encouraging, but further clinical studies in humans are needed to draw conclusions about the full potential for treating neurodegenerative diseases such as AD.

### 4.5. Diterpenoids

Diterpenes are terpenoids with four isoprene units which broadly exist in terrestrial and marine organisms [110]. Researchers have found that the compounds of the gracilins, structural analogue gracilin A (Figure 3H) in sponges can inhibit BACE-1, a kinase regulated by extracellular signals, and reduce hyperphosphorylation of tau protein [111], which has a neuroprotective effect on primary neurons [112]. In addition, they can also express strongly Nrf 2 involved in the activation of antioxidant pathway, produce important anti-inflammatory effects, and reduce the production of ROS induced by amyloid-β [88]. It can also induce targeting mitochondria to inhibit mitochondrial oxidation and play a neuroprotective role [113]. A study showed that Gracilin A could protect SH-SY5Y cells from hydrogen peroxidation-induced damage by reducing reactive oxygen species (ROS) levels, restoring glutathione (GSH) content, improving mitochondrial membrane potential (MMP) and increasing cell survival rate [114]. In a different study, the activity of Gracilin A derivatives was evaluated, including their ability to regulate antioxidant gene expression in SH-SY5Y cells and their anti-neuroinflammatory potential in LPS-activated BV2 microglia [115]. The results showed that Gracilin A can regulate the translocation of Nrf2 and NF-κB and reduce the activation of p38 mitogen-activated protein kinase (p38 MAPK) in SH-SY5Y and BV2 cells [115,116].In addition, it was also found that the abietane diterpenoids also have neuroprotective effects from *Phlegmariurus carinatus* [117]. Harziane Diterpenes from Deep-Sea Sediment Fungus *Trichoderma* sp. has a potential anti-inflammatory effect [117].

### 4.6. Alkaloids

Alkaloids are a kind of alkaline compounds containing nitrogen that exist in nature, mainly in plants, animals and algae. In the ocean, marine macrocyclic alkaloids mainly manzamines (Figure 3I), 3-alkylpiperidines, 3-alkyl pyridinium salts and so on [118]. Manzamine alkaloids are a class of complex β -carbonyl alkaloids isolated from spongy bodies with unique nitrogenous polycyclic systems [119]. It was found that the manzamine alkaloid is an inhibitor of glycogen synthetase kinase-3 beta (GSK3β) and reduces the activity of GSK3β through the hydrogenation of C-32/C-33 double bond and the oxidation of C-31 to ketone [67,120]. In cell experiments, manzamine A showed strong inhibition of tau phosphorylation in cells [67]. When the manzamines were evaluated in trials related to nervous system function and pathology, it did not show any effect on AChE or β-secretase, nor did it show significant ability to protect human neurocytoma SH-SY5Y cells against oxidative stress-induced cell death [121]. It is thus known that this alkaloid can be used to restrain the formation of NFTs by the hyperphosphorylation of tau protein, but has no effect on other clinical features of AD [122] Indole alkaloids (Figure 3J) are a kind of macromolecular compounds isolated from Streptomyces sp. In vivo, it has high activity for transcription of Nrf2 and has a neuroprotective effect [68]. Indoles have been confirmed to reduce tert-butyl hydroperoxide (TBHP)-induced cell death, demonstrating their neuroprotective potential and having little cytotoxic effect on human neuroblastoma SH-SY5Y and microglial BV2 cells [123]. It can be seen that this alkaloid can be considered to inhibit the occurrence of intracellular NFTs caused by tau hyperphosphorylation, but has no effect on other clinical features of AD, and is a potential drug for the treatment of AD. Alkaloids have been proven to inhibit Aβ plaque production and the hyperphosphorylation of tau protein, inhibit neuroinflammation and reduce apoptosis, activate autophagy, and reduce potential risk factors for AD, so they can have the potential to become a lead in AD treatment [124].

### 4.7. Amino Sulfonates

Amino sulfonate compound is a kind of compound matter that takes the place of hydroxyl group in sulfuric acid and belongs to the sulfur containing protein. For example, homotaurine (Figure 3K), obtained from various marine red algae, which is a kind of natural amino sulfonate compound. It has been confirmed that it has a protective effect on neurons in vitro and in vivo models in clinical studies [125]. The homotaurine reduces the toxic effects on neurons by encapsulating amyloid peptide to reduce misfolding and aggregation of amyloid to prevent the formation of amyloid-β oligomers and neurotoxic compounds [69]. The amyloid -β oligomer is a key pathogenic factor in neurological diseases and can form Aβ-fibrils and protofibrils that ultimately lead to the formation of amyloid plaques [70]. On the other hand, homotaurine has a similar structure to the neurotransmitter γ-aminobutyric acid (GABA), so it can specifically act on GABA receptors to reduce neurotoxic damage induced by glutamate delivery [126,127,128]. In a Phase II clinical trial, homotaurine has been shown to safely reduce the concentration of Aβ42 in cerebrospinal fluid (CSF) in patients with mild to moderate AD, contributing to the potential for improvement in the disease [116]. However, homotaurine was evaluated in phase III trials (ALPHASE trial) of mild-moderate AD and showed insufficient clinical efficacy [69]. The results showed a positive trend of Clinical Dementia Rating-Sum-of-boxes (CDR-SB), and the Alzheimer’s Disease Assessment Scale-cognitive subscale (ADAS-cog) results showed a statistically significant difference, but the planned analysis of the psychometric data showed no statistically significant difference [69]. Nevertheless, only the secondary endpoints of the study showed neuroprotective effect inhibiting Aβ activity of homotaurine. Therefore, further evidence is needed to determine the effectiveness of homotaurine in the improvement of AD, providing new possibilities for the treatment of AD.

### 4.8. Marine Toxins

Marine toxins are naturally toxic chemicals from the ocean environment [129]. Nereistoxin (NFX) was found to have a strong binding affinity with nicotinic acetylcholine receptor (nAChR) in mouse brain [130], as was Spirolides. Spirolides (SPX) are cycloimine lipophilic marine toxins, mainly produced by Alexandrium ostenfeldii (*A. ostenfeldii*) [71]. The toxicity of these compounds was detected for the first time in bivalve mollusks when mice that were intraperitoneally injected with scallop and mussel lipophilic extracts died unusually quickly, indicating a strong toxic reaction in the mice [131]. The mechanism of action of SPXs was not fully understood, but it had been proposed that cholinergic receptors are the main sites of action of these toxins [132]. It was non-toxic to humans and is an antagonist of nicotinic choline receptors, enhanceing the expression of the choline acetyltransferase enzyme (ChAT) [133]. The main representative substance was 13-desmethyl spirolide C (13-desMeC) (Figure 3L) which had been shown to reduce β-amyloid accumulation and reduce tau hyperphosphorylation through its action on cholinergic receptors in a three-transgenic mice (3xTg) neuron model [134]. This was due to the fact that the treatment of 3xTg cortical neurons with the toxin can observably decrease the levels of the hyperphosphorylated isoforms of tau recognized by AT8 and AT100 antibodies and the levels of intracellular β-amyloid [135]. It could also eliminate the glutamate-mediated neurotoxic effects, decreasing intracellular accumulation of Aβ and phosphorylated tau levels in neurons in vitro. Thus, it could be used for new treatment of AD in the body from the view of barrier of blood-brain [55]. Anatoxin a(s), a marine toxin originally extracted from filamentous cyanobacterium Anabaena flos-aquae, had been extensively studied for its AChE inhibition potential [136]. In vitro, the activity of AChE was determined by colorimetric assay. The results showed that anatoxin a(s) had a non-competitive inhibitory effect on AChE. These results were confirmed in vivo, where rats treated with anatoxin a(s) showed similar signs of anticholinesterase poisoning [137]. Therefore, marine toxins have the potential to treat AD by targeting certain targets, which requires further animal trails and clinical trials.

### 4.9. Macrolide-Type Compound

The macrolide caniferolide A (Figure 3M) is a kind of macrolide-type compound extracted from Streptomyces caniferus, a actinomycete in the ocean, with potential biological activity [138]. Caniferolide A have been confirmed to have neuroprotective effects [139]. Caniferolide A can reduce the content of neuroinflammatory markers in lipopolysaccharide-activated BV2 glial cells, block the transfer of FFKB-P65 to the nucleus and activate the Nrf2 pathway [138]. Rebeca Alvariño et al. [139] examined tau phosphorylation in SH-SY5Y tau441 cells and found that caniferolide A can reduce Thr212 and Ser214 phosphorylation by targeting p38 and c-Jun N-terminal kinase (JNK) mitogen activated kinases (MAPK). In addition, the antioxidant activity of macrolides was determined in the oxidative stress model of SH-SY5Y cells treated with H2O2. It was found that the compound could decrease the release levels of proinflammatory cytokines (IL-1β, IL-6 and TNF-α), ROS and NO, and increase the cell viability and GSH content. Finally, in order to confirm the anti-inflammatory effect of caniferolide A, BV2 microglia and SH-SY5Y neuroblastoma cell lines were trans-well co-cultured. The results showed that caniferolide A significantly increased the survival rate of neuroblastoma cells, confirming its neuroprotective properties [139]. All of these indicated that the compound could be effective and possibly used for novel AD treatment for AD.

Bryostatin 1 is a macrolide compound with high oxygen structure, isolated from the marine invertebrate Bugula neritina, which can be used as an effective regulator of protein kinase C (PKC) [72]. It can bind to PKC, cause quick short activation and autophosphorylation of PKCs, and induce continuous translocation of PKC membrane and continuous downregulation of PKC, leading to increased production and release of BDNF in the central nervous system [140]. Intraperitoneal injection of bryostatin 1 activates PKCε in the brain and prevents Aβ elevation, synaptic loss, and memory deficits in AD mouse model [141]. Preliminary safety and tolerability data for Bryostatin-1 in AD have been evaluated in phase IIa clinical trials (ClinicalTrials.gov identifier NCT02221947) with no serious adverse events and positive results [142]. Bryostatin concentration in the blood peaked one hour after the patient was infused. However, long-term treatment with bryostatin can induce downregulation of PKCε, depending on the duration and dose level of treatment. In another Phase II clinical study, bryostatin showed better efficacy, tolerability and safety when used to improve cognitive loss in 150 patients with advanced AD. However, the initial improvement was not significant in the full analysis set (FAS), and in the completer analysis set (CAS), primary and secondary analyses showed positive results in the bryostatin (20 μg) treatment group compared to the placebo group [143]. Therefore, these analyses collectively suggest that further trials are needed to evaluate the role of bryostatin in cognitive function in patients with AD. Therefore, in past few years, these compounds have been identified as promising drugs with potential.

## 5. Challenges and Opportunities in Developing Marine Natural Products for Alzheimer’s Disease

In 2018, the Alzheimer’s Disease International estimated that around 50 million people worldwide have dementia, a figure expected to triple by 2050, with two-thirds living in low-and middle-income countries [144]. Alzheimer’s disease has become a huge challenge the world is facing. However, unlike the rapidly growing number of Alzheimer’s patients, the types of drugs used to treat Alzheimer’s have not increased significantly. One of the reasons for the slow renewal of therapeutic drugs is the long clinical cycle of drugs. For instance, the phase 3 trial of Aducanumab (NCT05310071) lasted three years, and the phase Ⅲ trial of GV-971 lasted five years (NCT04520412). The second reason is the high failure rate of clinical trials. However, AD drugs have a high clinical failure rate, including several pharmaceutical giants (such as Pfizer, Roche, etc) [145]. Most clinical drug trials target at Aβ and tau, but the clinical failures of the recent decades indicate that there are further pathological mechanisms [146]. This prompted researchers to focus on the development of multi-targeted drugs [122,146,147]. Multi-target targeted ligands (MTDLs) strategy has been proposed and developed many times. The MTDLs design strategy involves the incorporation of distinct pharmacophores of different drugs in the same structure to get hybrid molecules [148]. The most widely used method is combining the structure of the FDA approved cholinesterase inhibitor with another drug with biological characteristics that is useful for treatment or combined using of several drugs. A new tacrine derivative with acetylcholinesterase inhibition (AChE) and brain-derived neurotrophic factor (BDNF) activation was obtained by linking tacrine with a fragment of huperzine A, and it has been shown to have a cognitive enhancement effect in two kinds of AD mice (APP/PS1transgenic mice and β-amyloid (Aβ) oligomers-treated mice) without inducing significant hepatotoxicity [149]. However, no relevant clinical trial information was found. A clinical trial (NCT01362686) involving a combination of three commonly drugs (donepezil, galantamine, rivastigmine) for AD was terminated due to a low study accrual.

The development of MTDLs also provides opportunities for the development of drugs with marine compounds as the main components. It can be seen from Table 2 that the targets for AD of most marine compounds are multidirectional rather than single. A single compound can act on multiple targets, which means that compared with synthetic drugs, the cumbersome drug synthesis process is eliminated. Secondly, marine compounds themselves are derived from edible natural organisms, many of which are edible compounds and have been widely used in food, cosmetics and other industries, indicating that their use safety is guaranteed [91,150,151]. At present, the biggest limitation of marine compounds to become clinical drugs is the lack of clinical trials. Most of the reported marine compounds with potential to treat Alzheimer’s disease remain in the animal experiment stage. As for the clinical progress of marine compounds mentioned in Section 4, only bryostatin (NCT02221947) and astaxanthin (NCT05015374) were found to have related clinical trials. Many marine compounds have not been put into clinical trials. This situation may be related to a lack of collaboration between different disciplines, especially within medical science. Analyzing the references, it was found that most of the authors who proposed marine compounds with potential to treat AD were involved in the chemical, biological, or marine sciences, and there was generally a lack of authors with medical backgrounds. The lack of support from medical professionals has greatly slowed the progress of marine compounds to the clinical platform. At present, the biggest limitation of marine compounds to become clinical drugs is the lack of clinical trials. Most of the reported marine compounds with potential to treat Alzheimer’s disease remain in the animal experiment stage. The Center for Disease Control and Prevention (CDC) could take the lead in drafting a protocol and recruiting medical researchers as well as chemical, biological, and marine researchers, which would accelerate clinical research of marine drugs. Another challenge for marine drugs is the stability and extraction of marine compounds. For example, marine indole alkaloids have many biological activities and their neuroprotective properties can be used in the treatment of AD. However, the determination of bioactivity has become a difficult problem due to the insufficient amount of isolated and purified compounds [68]. In another case, the yield of phlorotannins extracted from *Sargassum fusiforme* can only reach 6.36% [152]. Translating chemical diversity into pharmacological diversity is big problem, so the isolation and extraction of compounds in the drug application development process is big challenge [68]. It is gratifying that there is a lot of research focused on the green extraction of natural compounds [137,153,154,155]. In addition, food level nanocapsules, encapsulation technology and drug target delivery technology have gradually matured [156,157,158,159], which provide a strong technical guarantee for achieving transformation from marine compounds to marine drugs. So, there is no need to worry too much about this issue. With in-depth study, these problems can be solved. Furthermore, over the past decade, several efforts have been made to discover new biomarkers that could enable more accurate and rapid diagnosis of neurodegenerative diseases. These biomarkers include magnetic resonance imaging (MRI), which targets the cerebral cortex, white matter, etc., positron emission tomography (PET), which analyzes tau lesions and beta-amyloid accumulation, and cerebrospinal fluid (CSF) targeting β-amyloid polypeptides, β-amyloid oligomers, and tau peptides as well as blood biomarkers [160,161]. Biomarkers contribute to more rapid and accurate diagnosis, provide an indication of disease progression, and identify the best drug for a particular individual. Integrating these biomarkers into drug development or clinical trials for neurodegenerative diseases is an important step to help develop and demonstrate drug efficacy and target involvement [162]. The discovery of more drug targets will certainly encourage more research teams to explore new drugs, which also provides opportunities for marine compounds.

Therefore, it can be seen that marine resources offer us a huge library of potential research drugs. Marine natural molecules can be used as lead compounds for the development of drug candidates against AD. In order to ensure that drug development has a promising research progress and provide good opportunities and application prospects in the biomedical field, it is necessary to further exploration of molecular mechanisms, toxicity and side reaction of active ingredients, along with the development of in vivo and in vitro researches, are vital to the development of novel drugs for the treatment of AD, and we need describe their health implications [163]. In conclusion, using marine compounds for treating neurodegenerative diseases is both an opportunity and a challenge.

## 6. Remarks and Future Perspectives

This review examines the pathogenesis of Alzheimer’s disease in brief and emphatically introduced various marine compounds with the potential to treat Alzheimer’s disease. The literature shows that marine compounds are a cost-effective and environmentally friendly resource with high biomedical potential. Sodium oligomanne capsules (GV-971), an acidic oligosaccharide compound prepared from the extract of marine brown algae, was successfully applied in clinical practice, which also indicates the drug value of marine natural compounds. Nevertheless, the development of potential drugs is mostly undertaken by academics or small biotechnology laboratories. So, it requires the joint efforts and cooperation of researchers from all disciplines, the active advocacy of government departments, and the trust and cooperation between doctors and patients to accelerate the progress of marine compounds from laboratory to clinical trials. In addition, in order to search for anti-AD compounds of marine origin with significant neuroprotective activity and better understand the potential advantages of the characteristics of various marine organisms and their compounds for the healthy of people, it is necessary to encourage and stimulate more investment in biotechnology to contribute to the more sustainable development and use of these marine resources in the future.

## Figures and Tables

**Table 1:** Several related information about drugs currently used for AD treatment.

Drug’s Name	Chemical Structures	Pharmacological Mechanism	Ref.
Tacrine	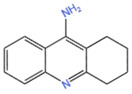	Inhibition of cholinesterase (both AChE and BChE)	[40]
Donepezile HCl	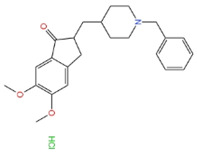	Inhibition of AChE	[41]
Rivastigmine	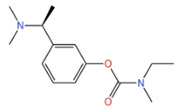	Reversible double inhibitors of AChE and BChE	[42]
Galantamine	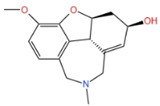	Weak competitive and reversible cholinesterase inhibitors	[38]
Memantine	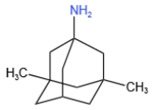	Antagonist of the NMDAR	[37]
sodium oligomannate	n = 1~9; m = 0, 1, 2; m’ = 0,1 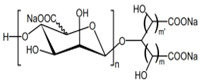	Directly combine with Aβ to reduce Aβ in brain deposition	[43]

**Table 2:** Main marine natural products with pharmacological activity to potentially treat AD.

Family	Compound	Origin	Pharmacological Activity	Ref.
Polysaccharide	Fucoidan	Brown seaweeds	Block caspase-9 and caspase-3 enzymes.	[57]
	Chitosan	Crustaceans	Inhibition of the enzyme acetylcholinesterase.	[58]
	Mannan	*Codium fragile*	Inhibition of the enzyme β-secretase (Bace1)	[59]
Polyphenol	Dieckol	Brown seaweeds (*Ecklonia cava, Ecklonia stolonifera*)	Inhibition of the enzymes acetylcholinesterase and Butyrylcholinesterase	[60]
Sterol	Fucosterol	brown alarge (*Panida australis*)	Anti-inflammatory and anti-BACE1	[61,62]
Carotenoid	Fucoxanthin	brown algae (*Sargassum siliquastrum*)	Anti-inflammatory and antioxidant	[63]
	Astaxanthin	green algae (*Haematococcus pluvialis*)	Decrease the production of NF-κB transcription factors and inflammatory cytokines	[64]
	Lycopene	Red seaweeds	Anti-inflammatory and antioxidant	[65]
Diterpenoid	Gracilins	Marine sponges (*Spongionella gracilis*)	Inhibition of the enzyme β-secretase or BACE-1.Anti-inflammatory and antioxidant properties Reduction in hyperphosphorylation of tau protein	[66]
Alkaloid	Manzamine	Marine sponges (Acanthostrongylophora)	GSK-3 inhibition and reduction in hyperphosphorylation of tau protein	[67]
	Indole	*Streptomyces* sp.	Inhibition Aβ plaque production by activating the nuclear factor Nrf2	[68]
Amino sulfonate	Homotaurine	Red seaweeds	Aβ lowering and prevention of the formation of a toxic soluble amyloid oligomer	[69,70]
Marine Toxin	Spirolides	*Alexandrium ostenfeldii*	Acetylcholinesterase inhibition, and restraint the formation of amyloid-β	[71]
Macrolide	Caniferolide A	*Phylum Actinobacteria*	Anti-inflammatory and antioxidant action Blockade of the BACE-1 enzyme.	[72]

**Table 3:** ADMET profiling of nine marine natural products.

Parameters ^1^	Fucoidan	Mannan	Fucosterol	Astaxanthin	Lycopene	Manzamine A	Indole	Homotaurin	13-desMeC
MW (130~725)	242.05	666.22	412.37	596.39	536.44	548.35	117.06	139.03	691.44
LogS (−4~0.5)	−0.042	0.762	−6.887	−7.226	−7.642	−3.86	−2.151	−0.14	−5.162
LogP (0~3)	−2.003	−4.868	7.447	8.045	11.072	5.459	2.292	−2.745	5.652
Pgp-inh	0	0	0.679	1	0.998	0.999	0.001	0.001	0.998
Pgp-sub	0.006	0.894	0.001	0.011	0.758	0.056	0.012	0.002	0.724
HIA	0.927	1	0.004	0.019	0.02	0.034	0.005	0.925	0.027
F (30%)	0.85	1	0.224	0.001	0.113	0.003	0.468	0.835	0.534
Caco-2	−5.53	−6.294	−4.624	−5.196	−5.708	−5.131	−4.259	−5.902	−4.813
BBB	0.678	0.45	0.818	0.001	0.001	0.99	0.737	0.936	0.25
PPB	18.86%	5.85%	98.64%	101.00%	99.44%	96.16%	86.32%	10.54%	97.60%
Fu	72.18%	54.83%	1.78%	2.56%	5.42%	3.27%	16.50%	87.62%	1.54%
CYP1A2-inh	0.003	0	0.058	0.015	0.266	0.28	0.975	0.005	0.005
CYP1A2-sub	0.171	0.002	0.436	0.156	0.521	0.786	0.805	0.237	0.817
CL	3.024	0.28	13.304	0.719	−0.286	7.851	11.189	3.782	18.565
T_1/2_	0.302	0.543	0.016	0.067	0.137	0.008	0.794	0.525	0.015
hERG	0.029	0.025	0.011	0.235	0.852	0.863	0.029	0.035	0.679
Ames	0.16	0.072	0.023	0.369	0.393	0.154	0.311	0.056	0.02
ROA	0.943	0.089	0.022	0.111	0.218	0.891	0.844	0.346	0.993
FDAMDD	0.027	0	0.638	0.974	0.948	0.932	0.219	0.014	0.942
BCF	0.444	0.105	3.317	1.465	2.187	1.081	0.934	0.212	1.755

^1^ MW: Molecular weight. LogS: The logarithm of aqueous solubility value. LogP: The logarithm of the n-octanol/water distribution coefficient. Pgp-inh: The inhibitor of P-glycoprotein. Pgp-sub: The substrates of P-glycoprotein. HIA: Human intestinal absorption. F (30%): The human oral bioavailability 30%. Caco-2: The permeability of human colon adenocarcinoma cell lines (Caco-2). BBB: the penetration of blood–brain barrier (BBB). PPB: Plasma protein binding. Fu: The fraction unbound in plasms. CL: The clearance of a drug. T1/2: The half-life of a drug. hERG: The human ether-a-go-go related gene. Ames: The Ames test for mutagenicity. ROA: The toxicity of rat oral acute. FDAMDD: The maximum recommended daily dose. BCF: The bioconcentration factor. The data acquired from ADMETLab 2.0 database.

## Data Availability

Not applicable.
